# Spatial Distribution of Heavy Metals and Pollution of Environmental Media Around a Used Lead-acid Battery Recycling Center in Ibadan, Nigeria

**DOI:** 10.5696/2156-9614-11.29.210304

**Published:** 2021-03-02

**Authors:** Elizabeth Oloruntoba, Olusegun Gurusa, Folashade Omokhodion, Julius Fobil, Niladri Basu, John Arko-Mensah, Thomas Robin

**Affiliations:** 1 Department of Environmental Health Sciences, University of Ibadan, Ibadan, Nigeria; 2 Department of Community Medicine, University of Ibadan, Ibadan, Nigeria; 3 Department of Biological, Occupational and Environmental Health, University of Ghana, Accra, Ghana; 4 Faculty of Agricultural and Environmental Sciences, McGill University, Montreal, Canada; 5 Department of Environmental Health Sciences, University of Michigan, Ann Arbor, USA

**Keywords:** used lead-acid battery, ULAB informal recycling, metals, environmental media, Ibadan, Nigeria

## Abstract

**Background.:**

Heavy metals are usually present in trace amounts in various environmental media such as water, soil, and air, and many are poisonous to human health even at very low concentrations.

**Objectives.:**

To assess the risk of heavy metal contamination of water, soil, and plants around a used lead acid battery (ULAB) recycling center in Ibadan, Nigeria.

**Methods.:**

Environmental samples (water, soil, and plants) were collected using standard methods and concentrations of arsenic (As), cadmium (Cd), antimony (Sb), chromium (Cr), copper (Cu), manganese (Mn), nickel (Ni), lead (Pb), selenium (Se), and zinc (Zn) were determined using inductively coupled plasma-optical emission spectrometry at the International Institute of Tropical Agriculture, Ibadan, Nigeria.

**Results.:**

The concentration of metals detected in water samples were higher than permissible limits at more than 50% of the sampling locations. In contrast, heavy metals in soil were within permissible limits. Most of the heavy metals except Pb were found to be present in the plant within permissible limits. Lead levels in water and plants from all locations exceeded the permissible limits. The contamination degree and pollution load index of water sources around the ULAB recycling center indicate a high degree of pollution of water sources with heavy metals, while soil samples were within the normal baseline levels. The transfer factor of Pb from soil to *Amaranthus viridis* was 1.92. This has implications for human health as the plant is often harvested and for sale in local markets as a source of food and medicine.

**Conclusions.:**

The present study recommends improved technology for ULAB recycling and adequate treatment of effluent/runoff from recycling centers before discharge.

**Competing Interests.:**

The authors declare no competing financial interests.

## Introduction

Lead-acid batteries (LAB) are used extensively “in storage technologies for renewable energy sources, e.g. solar cell and wind turbines; to power automobiles and other appliances (e.g. off-grid household electric power systems, portable televisions, etc.); hence it has been referred to as “a modern metal, supporting a modern world”.^1^ These are devices that store electrical energy with the help of cells made of lead alloys and an electrolyte (mixture of sulphuric acid and water). Official data compiled by the International Lead and Zinc Study Group (ILZSG) showed that as of 2012, the global production of lead (Pb) was 10.536 million tons, of which 55.04% was from secondary production from recycled products or residues arising from the production processes, while the global usage was 10.469 million tons.^2^ The World Health Organization (WHO) also reported that about 85% of the total global consumption of Pb is used for the production of LAB.^3^

Lead has been classified as a global environmental contaminant, while used lead-acid battery (ULAB) recycling and Pb smelting industries were ranked as the first and third of the ten worst polluting industries when ranked by disability-adjusted life years (DALYs).^4^ According to Ali *et al.*,^5^ contamination of biota and groundwater with potentially toxic heavy metals has important implications for human health. In Nigeria, heavy metal contamination of environmental media around informal ULAB recycling centers is a serious public health problem. There has been recent interest in the potential of solar panels and LABs for power generation. Increased use of LABs brings with it the public health and environmental costs of heavy metal contamination of air, water, soil and plants around ULAB recycling centers.^6^

Recycling of secondary Pb is normally done by the informal sector in many developing countries in a very crude way with the resultant release of high quantities of Pb into the environment.^7^ Exposure pathways include dermal, gastro-intestinal and inhalation from contamination of the air, soil and water sources (ground and surface) with resultant associated adverse health effects. A report credited to Pure Earth claimed that DALYs associated with adverse health impacts from Pb exposure at a site at a small town outside of Manila in the Philippines was 319 817 for the estimated exposed population of 15 000. 7 This is equivalent to 21 years lost or lived with a disability per person.^7^ A 2016 report published by Green Cross Switzerland and Pure Earth listed the top ten polluting industries based on each source's global burden of disease.^4^ Used lead-acid battery recycling was classed as number one followed by mining and ore processing, tanneries, dumpsites, industrial estates, smelting, artisanal small-scale gold mining (ASGM), product manufacturing, chemical manufacturing, and the dye industry. These industries collectively put over 32 million people at risk and account for 7 to 17 million DALYs in low- and middle-income countries of which ULAB recycling contributed between 2 and 4.8 million DALYs.^4^

**Abbreviations**
*FAO*: Food and Agriculture Organization of the United Nations
*ULAB*: Used lead acid battery
*WHO*: World Health Organization

Heavy metal pollution of the soil was reported by Gardea-Torresday *et al*.^8^ and Claus *et al.*^9^ to be one of the biggest environmental problems in the world. A greenhouse experiment by Adekunle *et al.*^10^ showed that Pb and cadmium (Cd) bioaccumulation in *Amaranthus cruentus L.* increased significantly with increasing levels of soil contamination, and the values were far above Food and Agriculture Organization (FAO)/World Health Organization (WHO) recommended safe limits. Dietary intake of heavy metals through contaminated vegetables may lead to various chronic diseases.^11^ In Nigeria, Dooyema *et al.* reported the deaths of children who presented with non-specific symptoms in two villages, Dareta and Yargalma in the Anka and Bukkuyyum local government areas (LGAs) of Zamfara State, northwest Nigeria.^12^ The incident was associated with acute Pb poisoning due to illegal mining of gold-rich ore. In addition, Olafisoye *et al.* reported the presence of significant concentrations of cadmium (Cd), chromium (Cr), zinc (Zn), Pb and nickel (Ni) in water, soils, and plants in e-waste dumpsites and residences in and around Alaba International Market, Lagos.^13^ In general, data on the activities of ULAB recyclers and the associated harmful environmental effects are scarce in low- and middle- income countries where ULAB recycling is conducted using simple but highly polluting techniques and processes. This study was designed to document the activities of ULAB recyclers and their impact on the receiving environment by assessing the levels of heavy metals in water, soil, and plants around the selected ULAB recycling center in Ibadan in Nigeria.

## Methods

The study was carried out in Ibadan, the capital city of Oyo State, southwest Nigeria in January 2018. Ibadan has an estimated population of 2 559 853. It covers a total area of 3 080 square kilometers.

**Figure 1:**
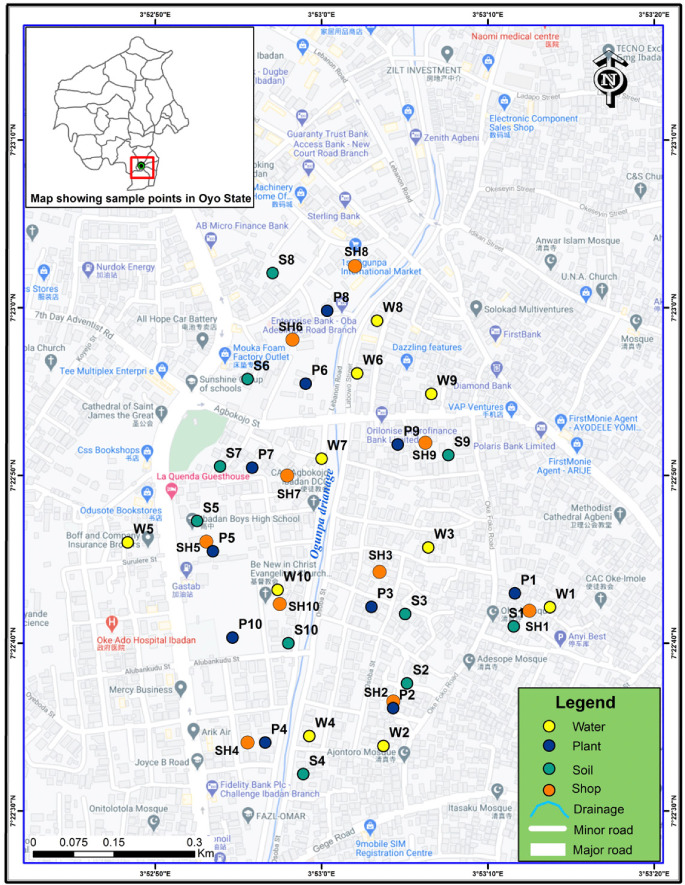
Map of Ibadan Southwest Local Government Area showing the sampling locations. (The letters W, P and S in the map represent water, plant, and soil samples, respectively; SH represents shops)

The study location was at Oke-Bola in Ibadan Southwest LGA informal ULAB recycling and smelting activities are pervasive. It has an area of 40 km^2^ and was estimated to have a population of 397 700 and a density of 9 943/km^2^ in 2016.^14^ It is one of the largest LGAs in Oyo State. The Local Government has its headquarters at Oluyole Estate. Its most populous areas or districts include Ring-Road, Oke-Ado, Oke-Bola, Isale-Osi, etc. There are no major farming activities in the area as it is primarily an urban center. Ibadan Southwest LGA is sub-divided into 12 wards. The Local Government is led by an elected chairman and twelve councilors, one elected from each ward. Figure 1 presents a map of the study area.

### Study design

A cross-sectional study design with a laboratory component was adopted to gather information on the level of heavy metal pollution in environmental media (water, soil, and vegetation/plants) arising from ULAB recycling activities in a selected center in Ibadan. Water, soil, and vegetation (plant) samples were collected from selected locations around the center. A global positioning system device was used to take coordinates of the sampling points.

### Sample collection procedures

Ten (10) locations were identified around the ULAB recycling center as shown in Figure 1. The site of the recycling center slopes towards the Ogunpa channel into which runoff from the center flows.

Polytetrafluoroethylene (PET) bottles were washed until thoroughly clean and rinsed with distilled water. Water samples were collected about 10 meters away from the river channel/drainage. Samples were collected in triplicates using standard methods from each of the 10 locations for a total of 30 samples. The samples were collected from wells, a nearby stream/river that receives run off from nearby houses and the recycling environment, and boreholes. Samples from the river channel and wells were collected from slightly below the surface of the water. However, the groundwater flow direction was unknown. The heavy metals in the water samples were preserved by adding nitric acid to minimize adsorption of the analytes onto the sample bottles.

#### Soil

The recycling center consists of makeshift shops which are located on both sides of the Ogunpa river channel/drainage. The site of the recycling center was steep and slopes towards the Ogunpa channel into which soil samples were collected from the topsoil at a depth of 0–15 cm by using a soil auger and stored in polythene bags labeled with paper tapes. Triplicate soil samples were collected from each of the 10 locations at the recycling center to make a total of 30 samples.

The soil was observed to be sandy in nature, but composition was not included in the analysis of the soil samples.

#### Vegetation

Vegetation grows indiscriminately in and around the recycling center, however, it was observed that vegetation was sparse at the point where battery terminals were being melted. Plants growing in and around the recycling center were collected using the techniques described by Radojevic and Bashkin^15^ and packed into plastic bags with a zip lock. A total of nine different plants were collected from five locations in the study area. The plants were identified and separated into leaves, stems, and roots. In all, a total of 30 samples were processed (3 per location).

### Extraction and analytical procedures

Concentrated nitric acid (HNO_3_) (2.5 ml) was added to 50 ml of acid preserved water sample and digested until a colorless solution was obtained. The digested samples were filtered to remove insoluble materials and the volume of the digested sample was made to 50 ml with distilled water in a volumetric flask.^16^ The distilled water was tested to ensure the absence of metals.

The soil samples were air-dried to preserve their characteristics. The digestion method by Francek *et al.*^17^ was adopted for the extraction of trace metals in the study. The soil samples were crushed and 1 g was accurately weighed and digested with 10 ml of 1:1 concentrated HNO_3_. The mixtures were evaporated to near dryness on a hot plate and cooled, and the procedure was repeated with 1:1 concentrated hydrochloric acid (HCl) (15 ml). The extracts were filtered with Whatman filter paper No. 40 and then made up to 100 ml with 2% HNO_3_.

The plants were washed with distilled water to remove debris and separated into parts (leaf, stem, and root). The partitioned plant parts were dried in an oven at 60ºC for 10 hours, then ground and powdered with a mortar. The powdered plant samples (1 g for each location) were digested using a mixture of concentrated nitric and perchloric acids.^16^

#### Determination of pH of water and soil samples

The pH of the water samples was determined using a Bosch PHS-25 CW pH meter. The electrode of the meter was rinsed with distilled water and blotted dry. The pH meter was calibrated first with a pH buffer and then with buffer 4. Then the sample was mixed and the electrode was placed in the sample while ensuring that the entire sensing edge was submerged. The pH values were recorded when the display on the meter was stable. A portion of the soil samples (20 g) was weighed into a 100-ml beaker, 20 ml of deionized water was added, and stirred for 30 minutes. The soil pH was determined using a pH meter.

#### Heavy metal determination

Concentrations of arsenic (Ar), Cd, antimony (Sb), Cr, copper (Cu), manganese (Mn), Ni, Pb, selenium (Se) and Zn in digested water, soil and plant samples were determined using inductively coupled plasma-optical emission spectroscopy (PerkinElmer Optima 8000, PerkinElmer, Inc., USA). Adequate quality assurance procedures were put in place.

### Data Analysis

All results from the field study were compiled and recorded in a prepared form. Data entered were analyzed using the Statistical Package for the Social Sciences (SPSS) software (IBM, SPSS 16.0, Chicago). Results were summarized using means and standard deviation. Metal concentrations were compared with guideline values (WHO guidelines^18^/Standard Organization of Nigeria (SON) standards,^19^ and the United States Environmental Protection Agency (USEPA)^20^ standards for water. For soil, various standards such as the WHO/FAO,^21^ EU guidelines (Ministry of Environment, Finland),^22^ Kun *et al.*,^23^ Department of Petroleum Resources, Nigeria,^24^ and Barceloux^25^ were considered for different variables. For plants, WHO^26^ and WHO/FAO standards^21^ were used as applicable. Pollution indices for metals in water and soil and transfer factors for metals from soil to plants were calculated as shown below.

### Determination of pollution indices

Previous studies (e.g. Muller,^27^ Qingjie et al.,^28^ and Weissmannová and Jifi^29^) have documented methods for estimating pollution indices. However, the method of Manoj and Padhy^30^ which utilized guideline values was adopted in this study as there were no background level reference or control data to use for the calculation. Pollution assessment indices such as contamination factor (CF), contamination degree (CD), and pollution load index (PLI) were used to assess the level of contamination of water and soil with metals, while transfer factor was used to assess the level of transfer of metals from soil to plants/vegetation around the recycling center.

#### Contamination factor

The contamination factor is the ratio between the content of each metal to the standard/guideline value (SGV) of each metal and can be calculated according to Equation 1. It allows a unitary assessment of soil pollution with a particular heavy metal at each location.


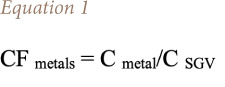


Table 1 shows the grading of the CF of a metal into four grades for monitoring the enrichment of a metal in soil using method by Islam.^31^

**Table 1:** Classification of Contamination Factor (CF) and Contamination Degree (CD) of a Metal

CF*	CD**	Description
CF < 1	CD < 7	Low degree of contamination
1 ≤ CF < 3	7 ≤ CD < 14	Moderate degree of contamination
3 ≤ CF < 6	14 ≤ CD < 28	Considerable degree of contamination
CF ≥ 6	CD ≥ 28	Very high degree of contamination

Source:

^*^Islam *et al.*^31^ and

^**^Hassan *et al.*^33^

### Contamination factor

Contamination degree allows the estimation of the degree of heavy metal pollution taking into consideration the “concentration of analyzed heavy metals in soils or individual values of the calculated indices such as the contamination factor”.^32^ The CD is determined using Equation 2.


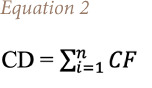


Table 1 shows the CD classification of a metal into four grades by Hassan *et al.*^32,33^

#### Pollution load index

Pollution load index was defined by Islam *et al.*^31^ as the n^th^ root of the multiplications of the CF of metals (*Equation 3*). In the present study, it gives an assessment of the overall toxicity status of the soil based on the contribution of eight out of the ten metals examined.


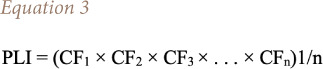


Classification of PLI values was adopted from Islam *et al.*, 31 such that:

PLI = 0: indicates no pollution
PLI = 1: indicates the presence of only baseline pollutant levels
PLI = >1: indicates pollution or progressive deterioration of the site and soil quality

According to Kowalska, *et al.*,^32^ CF provide information on the individual levels of pollution from each of the analyzed heavy metals, while CD and PLI give the scale of total pollution.

#### Transfer factors (TF) for heavy metals from soil to plants

The transfer factor is the ratio of the concentration of heavy metals in a plant to the concentration of heavy metals in soil. It is a measure of the uptake (accumulation) of metals by plants or vegetation around the ULAB recycling site. The TF for each heavy metal was computed based on the method described by Harrison and Chirgawi 34 according to Equation 4.

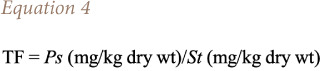



Where:

*Ps* is the plant-metal content originating from the soil and *St* is the total heavy metal contents in the soil.

A TF >1 indicates uptake of the specified metal in the plants.

## Results

Recycling of ULABs is conducted by crude processes capable of causing environmental degradation and poses a human health risk. The center in the present study has clusters of small make-shift shops where recyclers and their apprentices manually break open the batteries and the liquid (acid) in them is discharged into the surrounding environment.

### The pH of water and soil samples across locations

**Figure 2:**
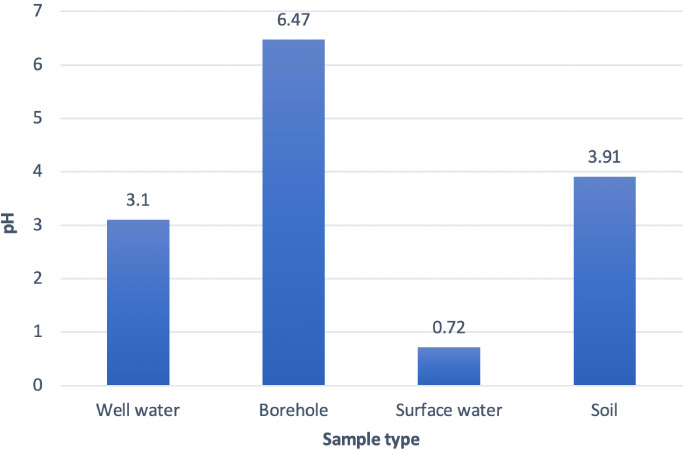
pH values of water and soil samples

The pH values for most of the water samples were far below the permissible limit except for samples from boreholes. The mean pH of the water samples from the 10 locations was 3.29±2.50 with a minimum of 0.59 from one of the samples from the stream/river to the highest of 6.54 in one of the boreholes. The recyclers claimed that water from the wells was only used for washing and were not a source of drinking water. Soil samples also had low pH values *(Figure 2);* the mean pH was 3.91±1.99 with a range of 2.13 to 6.74. The low pH values in the soil at the time of data collection may be due to sulfuric acid from the ULAB being discharged into the surrounding environment. Table 2 shows the mean pH values of water and soil samples from different locations in the recycling center.

**Table 2:** Water and Soil Sample pH Values Across Locations in the Recycling Center

**Locations**	**pH (Water)**	**pH (Soil)**
L1 (W)	2.99±2.76	2.44±0.83
L2(W)	5.86±0.41	2.66±1.47
L3(W)	1.08±0.48	2.35±0.17
L4(W)	1.34±0.23	2.71±0.50
L5 (B)	6.39±0.48	6.74±0.29
L6 (B)	6.54±0.65	4.68±2.86
L7 (W)	1.60±1.28	6.29±0.36
L8 (W)	5.70±1.01	5.96±0.24
L9(R)	0.59±0.21	2.13±0.44
L10 (R)	0.84±0.28	2.74±0.56
Min	0.59	2.13
Max	6.54	6.74
Median	2.295	2.725
Mean	3.29±2.50	3.91±1.99
EPA Limit^20^	6.5–8.5	
SON Limit^19^	6.5–8.5	
WHO/FAO^21^		6.5–8.5
Limit		

Key: W = well, B= borehole, R= river, Lx = locations

Note: Orange text signifies pH values below permissible limits.

### Concentrations of heavy metals in water samples

Table 3 presents the mean concentrations of 10 different heavy metals in water samples collected from 10 locations around the recycling center. Mean values were 0.19±0.06, 0.29±0.08, 1.64±1.20, 6.17±3.95, 15.73±22.18, 6.28±8.91, 1.34±0.66, 2.88±3.64, 0.03±0.01, 25.80±28.44 mg/l for As, Cd, Sb, Cr, Cu, Mn, Ni, Pb, Se and Zn respectively. These values were above the permissible limits except for selenium for all the metals. The concentration of metals detected in water samples were higher than permissible limits at more than 50% of the locations. Lead levels in samples from all the locations exceeded the permissible limits. The mean concentrations of heavy metals followed a decreasing order of Zn>Cu>Mn>Pb>Cr>Sb>Ni>Cd>As>Se.

**Table 3:** Concentration of Heavy Metals in Water Samples from Selected Sites at the Used Lead-Acid Battery Recycling Center

Metals (Mg/L)	LI (w)	L2 (w)	L3 (w)	L4 (w)	L5 (B)	L6 (B)	L7 (w)	L8 (w)	L9 (R)	L10 (R)	USEPA^20^	SON^19^	WHO^18^
As	N/D	0.17	0.23	0.2	N/D	0.18	0.13	0.24	0.18	N/D	0.01	0.01	0.01
Cd	N/D	N/D	0.35	0.39	N/D	0.02	0.12	N/D	0.7	0.17	0.005	0.003	0.03
Sb	N/D	N/D	2.73	2.84	N/D	N/D	0.33	N/D	0.24	1.21	0.006	0.005	0.02
Cr	N/D	N/D	8.96	9.38	0	0.01	1.58	N/D	8.15	6.89	0.1	0.05	0.05
Cu	0.13	0.06	28.27	30.5	0.14	0.27	7.21	0.1	60.34	30.29	1.3	1	2
Mn	0.67	0.54	5.21	8.18	0.94	1.06	6.25	0.65	14.62	22.94	0.05	0.2	0.4
Ni	N/D	N/D	1.7	1.77	N/D	N/D	0.27	N/D	1.57	1.4	0.1	0.02	0.07
Pb	0.69	0.88	1.4	1.15	1.49	3.7	8.8	0.12	4.06	4.21	0.015	0.01	0.01
Se	0.04	0.04	N/D	N/D	0.04	0.02	N/D	0.04	N/D	N/D	0.05	0.01	0.04
Zn	0.39	0.19	70.89	72.04	0.75	8.55	13.39	0.44	48.46	36.06	5	3	3

Note: Orange text signifies permissible limit exceedances

### Concentrations of heavy metals in soil samples

Table 4 details the concentration of heavy metals in soil samples collected from 10 locations. Arsenic and Cd were not detected at any of the locations. The mean concentrations of Sb, Cr, Cu, Mn, Ni, Pb, Se, and Zn were 0.09±0.14, 0.09±0.12, 4.09±5.93, 1.47±1.71, 0.56±0.86, 2.25±0.90, 0.02±0.01, and 10.28±10.13 mg/kg, respectively. The mean concentrations of most of the parameters were below permissible limits (WHO/FAO)^21^ and European Union (EU).^22^ The mean concentrations of metals in the soil samples followed a decreasing order of Zn>Cu>Pb>Mn>Ni>Sb=Cr>Se.

**Table 4:** Mean Concentration of Heavy Metals in Soil Samples Collected Around the Used Lead-Acid Battery Recycling Center

Metals Mg/kg	L1	L2	L3	L4	L5	L6	L7	L8	L9	L10	Limits
As	N/D	N/D	N/D	N/D	N/D	N/D	N/D	N/D	N/D	N/D	5*
Cd	N/D	N/D	N/D	N/D	N/D	N/D	N/D	N/D	N/D	N/D	3†
Sb	N/D	N/D	N/D	N/D	0.18	0.07	N/D	0.01	N/D	N/D	2*
Cr	0.08	N/D	N/D	N/D	0.04	0.12	0.22	0.02	0.21	0.14	100†
Cu	1.9	0.1	0.85	0.39	8.86	3.83	12.49	8.74	0.2	1.69	100†
Mn	0.14	0.06	0.44	0.1	1.76	2.92	3.19	1.13	0.75	3.79	330***
Ni	2.08	N/D	N/D	0.02	N/D	N/D	0.28	N/D	N/D	0.21	50†
Pb	1.51	2.28	1.58	2.08	2	2.31	1.89	2.3	3.41	2.83	50†
Se	0.03	0.02	0.02	0.02	0.02	0.01	0.01	N/D	N/D	N/D	0.4**
Zn	5.06	7.16	9.23	7.11	9.12	14.21	8.04	7.13	4.95	31.89	300†

^†^WHO/FAO^21^

^*^ EU Guideline (Ministry of the Environment Finland)^22^

^**^ Kun *et al.*^23^

^***^Department of Petroleum Resources^24^ and Barceloux^25^

N/D = Not detected

### Types of plants collected in and around the recycling center

Nine different plants were collected from five locations in the study area. These included *Monechma ciliatum, Tridax procumbens, Amaranthus viridis, Acalypha ciliata, Solenostemon monostachyus, Scoparia dulcis, ferns, Kyllinga sp.,* and *Microgramma owariensis*.

In many communities, including those around the recycling center, traditional livestock management practice is characterized by a free-range system.^35^ It is also common to see goats, sheep, cows, and chicken grazing (feeding on plants) along the roads and around shops in the study location.

Many of the plants are weeds, such as *Monechma ciliatum* which has been described as a weedy herb. It is used for herbal medicine or as fodder for animals.^36^ According to Gusau *et al.*,^37^ *Monechma ciliatum* is traditionally used to treat different diseases such as body pain, liver, cold, diarrhea and sterility in women. The names, uses, and photographs of the plants can be seen in Supplemental Material.

### Concentration of heavy metals in plant samples

The mean concentrations of heavy metals in plants were 0.37±0.00, 0.09±0.11, 0.36±0.89, 0.30±0.34, 2.47±1.12, 0.01±0.01, 1.59±2.65 mg/kg for Sb, Cr, Cu, Mn, Pb, Se and Zn, respectively *(Table 5).* Arsenic, Cd, and Ni were not detected in all the samples at any of the locations. Antimony was detected in only one plant, while Pb had mean concentrations greater than the permissible limit (WHO/FAO) in all the locations. The mean concentrations of metals in the plant samples followed the decreasing order of Pb>Zn>Mn>Sb>Cu>Cr>Se.

**Table 5:** Mean Heavy Metal Concentrations in Plant Samples Collected Around the Recycling Center

Metals Mg/kg	L1	L2	L3	L4	L5	L6	L7	L8	L9	L10	WHO/FAO^21^	WHO^26^
As	N/D	N/D	N/D	N/D	N/D	N/D	N/D	N/D	N/D	N/D		0.1
Cd	N/D	N/D	N/D	N/D	N/D	N/D	N/D	N/D	N/D	N/D	0.1	0.02
Sb	N/D	N/D	N/D	N/D	0.37	N/D	N/D	N/D	N/D	N/D		
Cr	N/D	N/D	N/D	N/D	0.02	N/D	N/D	0.17	N/D	N/D	1.36	1.3
Cu	0.2	0.13	0.38	0.1	2.06	0.12	0.29	0.13	0.1	0.08	73	10
Mn	0.12	0.12	0.3	0.15	0.43	1.22	0.21	1.1	0.23	0.2	500	
Ni	N/D	N/D	N/D	N/D	N/D	N/D	N/D	N/D	N/D	N/D	67	10
Pb	2.72	3.48	3.03	2.72	2.68	2.15	2.06	1.72	2.2	1.97	0.3	2
Se	N/D	N/D	0.01	0.01	N/D	N/D	N/D	N/D	N/D	0.01		
Zn	2	2.13	1.51	6.54	1.43	0.4	0.16	0.41	0.25	0.09	100	0.6

Orange text signifies permissible limit exceedances.

### Assessment of metal pollution of water sources around the recycling center

**Figure 3:**
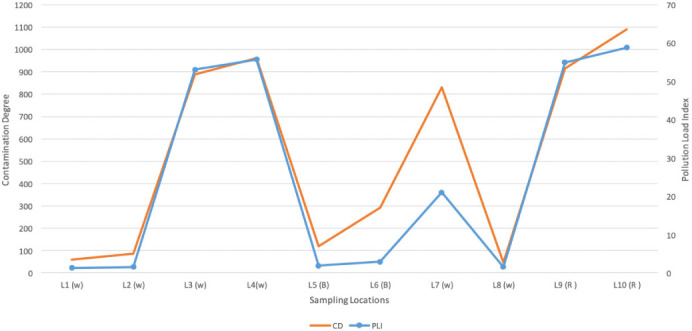
Contamination degrees and pollution indices of water samples across study locations

All 10 locations showed very high degree of contamination for Mn and Pb with CF ≥ 6, while in 7 locations the concentration of As was CF>6. In all, 41% and 54% of the locations showed a low and very high degree of contamination, respectively *(Table 6).* This study showed very high CD 50% of the locations. Lead levels in samples from all the locations exceeded the permissible limits. The mean concentrations of heavy metals followed a decreasing order of Zn>Cu>Mn>Pb>Cr>Sb>Ni>Cd>As>Se. (CD>28) and PLI>1 which indicate progressive deterioration of the water sources in the study area as seen in Table 7. Figure 3 details the CD and PLI of the water samples at different locations.

**Table 6:** Contamination Factor of Metals in Water Samples

Locations	L1 (w)	L2 (w)	L3 (w)	L4 (w)	L5 (B)	L6 (B)	L7 (w)	L8 (w)	L9 (R)	L10 (R)
As	ND	17	23	20	ND	18	13	24	18	ND
Cd	ND	ND	70	78	ND	4	24	ND	140	34
Sb	ND	ND	456.17	474.33	ND	ND	55	ND	40	202.5
Cr	ND	ND	89.6	93.8	ND	0.1	15.85	ND	81.5	68.9
Cu	0.1	0.05	21.75	23.46	0.11	0.21	5.55	0.08	46.42	23.3
Mn	13.4	10.84	104.2	163.6	18.8	21.32	125	13.04	292.4	458.8
Ni	ND	ND	17.02	17.75	ND	ND	2.74	ND	15.7	14.02
Pb	46	58.67	93.47	76.67	99.33	246.67	586.67	8	270.67	280.67
Se	0.8	0.8	ND	ND	0.8	0.4	ND	0.8	ND	ND
Zn	0.08	0.04	14.18	14.41	0.15	1.71	2.68	0.09	9.69	7.21

**Table 7:** Pollution Load Indices (PLI) and Contamination Degree (CD) of Metals in Water and Soil Samples

		LI	L2	L3	L4	L5	L6	L7	L8	L9	L10
Water	PLI	1.311	1.586	53.112	55.775	1.904	2.937	21.051	1.549	54.982	58.852
	CD	60.378	87.393	889.377	962.020	119.195	292.411	830.481	46.007	914.374	1089.4
Soil	PLI	0.010	0.0064	0.014	0.005	0.020	0.019	0.017	0.008	0.007	0.014
	CD	0.184	0.1208	0.122	0.120	0.306	0.203	0.233	0.166	0.091	0.198

### Assessment of metal pollution of soil around the ULAB recycling center

**Figure 4:**
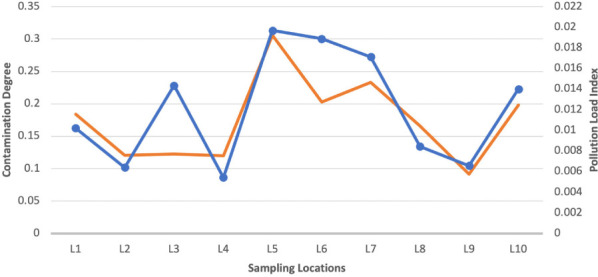
Contamination Degrees and Pollution Indices of Soil Samples Across Study Locations

Tables 7 and 8 present the contamination factors for each of the heavy metals that were detected in the soil samples from the different locations. Results showed CF<1 for all the metals, indicating a low degree of contamination. Figure 4 details contamination degrees and pollution load indices of soil samples at the different locations with CD<7, indicating a low degree of contamination and PLI <1 which indicates the presence of a baseline level of pollutants. The pattern of variation of CD and PLI are the same. Contamination degree showed a sharp decrease at location 6 and increased thereafter; however, the trend for PLI was slightly different.

**Table 8:** Contamination Factor of Metals in Soil Samples

	L1	L2	L3	L4	L5	L6	L7	L8	L9	L10
Sb	ND	ND	ND	ND	0.0910	0.035	ND	0.0050	ND	ND
Cr	0.0008	ND	ND	ND	0.0004	0.0012	0.0023	0.0002	0.0021	0.0014
Cu	0.0190	0.0010	0.0085	0.0039	0.0886	0.0383	0.1249	0.0874	0.0021	0.0169
Mn	0.0005	0.0002	0.0015	0.0003	0.0059	0.0097	0.0106	0.0038	0.0025	0.0126
Ni	0.0416	ND	ND	0.0004	ND	ND	0.0056	ND	ND	0.0042
Pb	0.0302	0.0458	0.0317	0.0416	0.0400	0.0462	0.0378	0.0460	0.0682	0.0566
Se	0.0750	0.0500	0.05	0.0500	0.0500	0.0250	0.0250	ND	ND	ND
Zn	0.0169	0.0239	0.0308	0.0237	0.0304	0.0474	0.0268	0.0238	0.0165	0.1063

^*^As and Cd were not detected, hence not included in the Table

### Transfer factors for heavy metals from soil to plants

Table 9 shows the rate of transfer of metals from soil to plants in the study locations. A transfer factor (TF) greater than one indicates uptake of the specified metal in the plants. In the present study, Sb with a factor of 2.06 for the *Tridax procumbens* plant had the highest TF, followed by Pb for *Amaranthus viridis* with a transfer factor of 1.92. Amarantus (from other locations), *Tridax procumbens*, fern, and *Solenostemon monostachyus* were also found to uptake Pb and other metals (Cr and Mn) from the soil as their TF values were greater than one. *Amaranthus viridis* is often harvested and sold in local markets as a source of food and medicines. It is able to accumulate metals and therefore has implications for human health.

**Table 9:** Transfer Factors of Metals from Soil to Plants

Locations	Plant name	Sb	Cr	Cu	Mn	Pb	Se	Zn
L1	Fern	*nil*	*nil*	0.11	0.86	1.80	*nil*	0.40
L2	*Amarantus spp.*	*nil*	*nil*	1.3	2	1.53	*nil*	0.30
L3	*Amarantus spp.*	*nil*	*nil*	0.45	0.68	1.92	0.5	0.20
L4	*Amarantus spp.*	*nil*	*nil*	0.26	1.5	1.31	0.5	0.92
L5	*Tridax procumbens*	2.06	0.5	0.23	0.24	1.34	*nil*	0.16
L6	*Acalypha ciliate*	*nil*	*nil*	0.03	0.42	0.93	*nil*	0.03
L7	*Solenostemon monostachyus*	*nil*	*nil*	0.02	0.07	1.10	*nil*	0.02
L8	*Kyllinga specie*	*nil*	0.5	0.02	0.09	0.75	*nil*	0.06
L9	*Monechma ciliatum*	*nil*	*nil*	0.5	0.31	0.65	*nil*	0.05
L10	*Scoparia dulcis*	*nil*	*nil*	0.05	0.05	0.70	*nil*	0.003

### Relationship between pH and metals in water, soil, and plant samples

**Figure 5:**
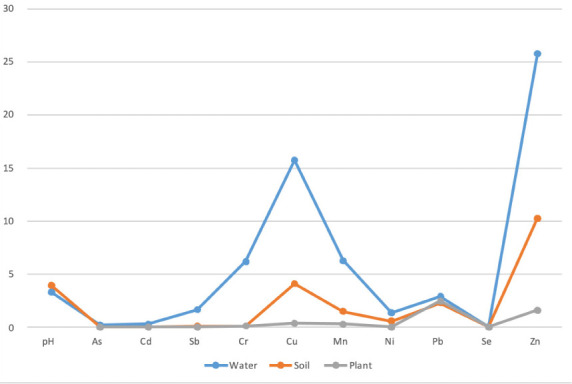
Mean concentrations of metals in water, soil, and plants

Figure 5 presents the mean concentrations of metals in water, soil and plants samples collected around the recycling center.

### Correlation of pH with heavy metals in soil and water samples

The correlation analysis of pH and heavy metals in water and soil samples is presented in Tables 10 and 11, respectively. Values with single (*) and double (**) asterisks show correlations at 0.05 and 0.01 levels of significance, respectively. From the matrix in Table 8, there was a significant negative correlation between pH of water samples and heavy metals present such as Cr (r = −0.677, P<0.01), Cu (r = −0.446, P<0.01), Mn (r = −0.472, P<0.01), Ni (r = −0.496*, P<0.05), and Zn (r = −0.594, P<0.01). On the other hand, as shown in Table 9, the correlation matrix of the soil samples revealed that of all the heavy metals analyzed, only Cu had a positive correlation with soil pH (r = 0.592, P<0.01).

**Table 10:** Correlation Matrix of pH and Heavy Metal Concentrations of Water Samples

Metals	pH	As	Cd	Sb	Cr	Cu	Mn	Ni	Pb	Se	Zn
pH	1										
As	−0.01	1									
Cd	−0.333	−0.08	1								
Sb	−0.425	0.420	−0.158	1							
Cr	−0.677**	−0.677	0.373	0.228	1						
Cu	−0.446**	0.233	0.380	0.049	0.806**	1					
Mn	−0.472**	−0.034	−0.011	−0.066	0.583**	0.770**	1				
Ni	−0.496*	0.787	0.145	0.400	0.986**	0.654**	0.330	1			
Pb	−0.244	−0.545	−0.156	−0.249	−0.042	−0.065	0.163	−0.341	1		
Se	−0.020	0.277	0.000	−7.21	0.000	−0.202	−0.359	0.000	−0.203	1	
Zn	−0.594**	0.258	0.466	0.818**	0.903**	0.700**	0.539**	0.733**	0.031	−0.125	1

Note: ^*^ Correlation significant at p<0.05

^**^ Correlation significant at p<0.01

**Table 11:** Correlation Matrix of pH and Heavy Metal Concentrations of Soil Samples

Metals	pH	Sb	Cr	Cu	Mn	Ni	Pb	Se	Zn
pH	1								
Sb	0.332	1							
Cr	−0.128	−0.482	1						
Cu	0.592**	−0.341	0.452	1					
Mn	0.227	−0.206	0.647*	0.684**	1				
Ni	−0.340	0.000	1.000**	−0.220	0.4351	1			
Pb	-	-	0.670	0.140	0.411*	10.368	1		
Se	-	-	0.000	-	-	0.000	−0.162	1	
Zn	-	0.227	0.439	0.056	0.584**	−0.449	0.418	−0.149	1

Note: ^*^ Correlation significant at p<0.05

^**^ Correlation significant at p<0.01

## Discussion

Generally, the mean pH values of water and soil samples collected from various locations around the ULAB recycling centers show they were acidic in nature. The acidic nature of both water and soil samples could be attributed to various battery recycling processes including discharge of sulfuric acid into the surrounding area without regard to the environment and thereby likely contaminating the soil. The contamination of water sources around the center may be the result of run off from the recycling center or through percolation through the soil into the water table. Low pH could affect the adsorption rate of heavy metals in both water and soil. This is the assertion of Appel and Ma^38^ who reported a low pH value as the main factor affecting heavy metal adsorption characteristics. It was also suggested that low pH could affect the solubility of the carbonates, hydroxides, and phosphates of heavy metals.

Concentrations of heavy metals in the water samples collected from the ULAB recycling center across the ten sampling locations in the present study were higher than permissible limits set by both the USEPA and SON at more than 50% of the locations except for selenium, which was within permissible limits. Heavy metals contamination of ground and surface water results in deterioration of water quality with the possibility of bioaccumulation along the food chain, and risks to human health. Various studies have reported the pollution of water sources with heavy metals with the possibility of creating adverse effects on riverine ecosystems and health.^39,33^ Nguyen *et al.* also reported that certain metals, including Pb, Cr, Cd, Hg, and As are highly toxic to aquatic and human life, causing kidney and liver problems in addition to genotoxic carcinogens.^40^

Findings from the analysis of heavy metal concentrations in soil samples revealed that the concentration of most metals at various sampling locations around the ULAB recycling center were within the permissible limits set by the WHO, FAO, and the European Union. A study conducted by Opaluwa *et al.* around a dump site in Lafia metropolis in Nasarawa State concluded that concentrations of heavy metals were within the permissible limits, except for Pb which was found to exceed the limit.^41^ However, a study of soil pollution around e-waste recycling operations in Ibadan by Adesokan *et al.* reported gross pollution of soil samples with metals in amounts that exceeded background concentrations by many folds.^42^ The low concentration of Pb in the soil samples in the present study may be a result of low pH values which could influence solubility of metals. This study is therefore in agreement with the findings of Appel and Ma which reported that soil pH plays a major role in the sorption of heavy metals and control of the solubilities of metal hydroxides, carbonates and phosphates.^38^ They also reiterated the possibility of soil pH affecting metal hydrolysis, ion-pair formation, and organic matter solubility. This may result in the soil having low affinity for metals, hence the low concentrations obtained in this study. Quoting from the work of McBride^43^ on the effect of pH on the affinities of tropical soil for heavy metals, Appel and Ma^38^ affirmed that an increase in soil pH can increase cationic heavy metal retention to soil surfaces via adsorption, inner-sphere surface complexation and/or precipitation, and multinuclear type reactions. This may be responsible for the low concentration of metals in the soil samples in the present study. In addition, Zhang *et al.* affirmed that pH is one of the main factors affecting the speciation of heavy metals and their subsequent migration and transformation in soil.^44^ For instance, a study on sorption characteristics of Cd and Pb in three tropical soils at varying metal concentrations and pH values concluded that Pb was sorbed more strongly than Cd in the soils and posed less of a threat to underlying ground water systems due to its lower mobility and availability.^38^

This study also revealed that seven (Sb, Cr, Cu, Mn, Pb, Se and Zn) out of ten heavy metals analyzed were detected in plants, although most of the metals were within the permissible limits except for Pb. A similar study reported by Oluyemi *et al.* also confirmed the presence of heavy metals in plants around a landfill area, although within the permissible limits.^45^ However, a study on heavy metal concentrations in soil and vegetation at the Korle Lagoon area of Accra by Fosu-Mensah *et al.* concluded that the concentrations of Cu, Pb, Ni and Cd in the plant samples collected from their study sites exceeded WHO/FAO permissible limits.^46^ Similarly, Zhou *et al.* affirmed the ability of leafy vegetables to uptake and accumulate heavy metals. The elevated level of Pb in plants found in this study could have been due to the activities in and around the ULAB recycling center.^47^ Consumption of vegetables growing around this center (especially *Amaranth*) could pose serious health threats to humans and grazing animals. Sultana *et al.* computed a health risk assessment of the consumption of contaminated vegetables from industrial areas using USEPA human health risk assessment models.^48^ They concluded that average heavy metal concentrations were higher in leafy vegetables compared to non-leafy vegetables and fruits, while risks from carcinogenic heavy metals in the studied vegetables and fruits were attributable to Cd (81%) > As (17%) > Pb (2%). However, in the present study, some of the weeds found at the recycling center (apart from *Amaranth* which is a common leafy vegetable) could be good candidates for phytoremediation of the site.

The analysis of the CD and PLI of water sources around the ULAB recycling center indicate a progressive deterioration of water sources around the center. These findings are in contrast to those reported by Bentum where the PLI calculated for five study sites indicated that the lagoon was practically unpolluted with heavy metals such as Fe, Cu and Zn, but moderately polluted with Pb and Ni.^49^ The reason for the difference in the PLI could be a result of the recycling of ULAB being practiced in the study location. The CD and PLI of the soil samples only indicate a low level of heavy metal contamination. This agrees with a study by Jorfi *et al* where heavy metals in soil were found to have only a baseline level of the pollutants.^50^

The transfer factor of most of the metals to plants were within the normal range except for Sb and Pb, which were found to have been transferred to plants such as *Amarantus, Tridax procumbens,* and *Solenostemon monostachyus*. Certain plants are known to have the capacity to accumulate heavy metals from contaminated soil, hence their detection in plants was not surprising. Continual consumption of plants contaminated by heavy metals could lead to accumulation and adverse health effects.^51^ This study is consistent with the findings of Oluyemi *et al.* which reported high transfer factors for As, Cd, Pb in *T. triangulare*, a vegetable in Nigeria.^45^

Findings from the correlations of pH with various heavy metals found in the water samples show that the pH of the water samples had a negative correlation with heavy metals such as Cr, Cu, Mn, Ni, and Zn. This shows that as the pH of the water decreases (acidic), the concentration of metals in water increases. pH has been reported to be one of the factors that could influence heavy metal distribution, speciation, and migration.^52^ Fondriest Environmental, Inc.^53^ reported that low pH levels can encourage the solubility of heavy metals and that metals such as Al, Cu, Pb and Cd would tend more to be released into water as the level of hydrogen ions increases, rather than get adsorbed into sediment. This may explain the high concentration of some metals in water, but low concentrations in soil samples. The correlations from the soil samples only found Cu to be positively correlated with pH. This indicates that as the pH increases, the concentration of Cu will continue to increase in soil.

## Conclusions

The present study found the pH of both water and soil samples to be acidic, which could influence the distribution, migration, and solubility of heavy metals. The study also found heavy metal concentrations in water samples at more than 50% of the locations to be higher than permissible limits. On the other hands, heavy metals in soil were within the permissible limits. Heavy metals were found to be present in plant samples but within the permissible limits except for Pb. The CD and PLI of water sources around the ULAB recycling center indicate a high degree of pollution of water sources around the center with heavy metals while soil samples were within the normal baseline for pollutants. The transfer factor analysis showed that Sb and Pb have been accumulated by plants such as *Amarantus, Tridax procumbens*, and *Solenostemon monostachyus*. The presence of Pb in *Amarantus* has implications for human health as the plant is often harvested and sold in local markets as a source of food while others such as *Solenostemon monostachyus* are used for medicinal purposes. The correlation analysis in water samples found pH to be negatively correlated with certain metals, while only Cu was found to be positively correlated with pH. The study recommends improvement in the technology for ULAB recycling and adequate treatment of effluent/runoff from recycling centers before discharge. Future studies should include soil sampling from various depths as this may provide more information on the speciation, mobility and leaching of Pb in the soil and comparison with results from centers in other locations/local government areas.
