# Supplementary material for: Spatial Distribution of Heavy Metals and Pollution of Environmental Media Around a Used Lead-acid Battery Recycling Center in Ibadan, Nigeria
Source: J Health Pollut. 2021 Mar 2;11(29):210304. doi: 10.5696/2156-9614-11.29.210304 (PMC8009648; doi:10.5696/2156-9614-11.29.210304)
Supplement: Supplementary file 1 [file Oloruntoba_Supplemental_Material.docx]

**Supplemental Material**

**Plants growing in and around the ULAB recycling center
Plants collected from the study location**

|  | Plant name | Photograph | Uses | References |
| --- | --- | --- | --- | --- |
| 1 | *Monechma ciliatum* | 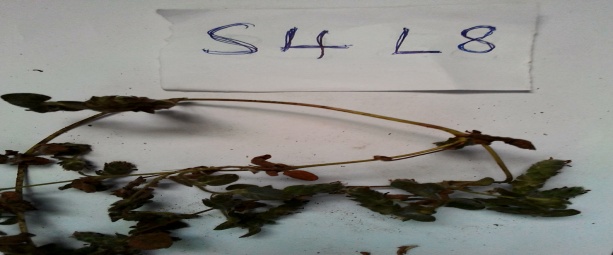 | -Weed  -Herbal medicine  and fodder | Gusau *et al.***^1^** |
| 2 | *Tridax procumbens* | 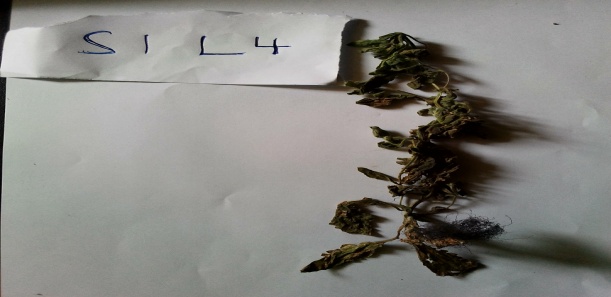 | -Weed  -Herbal medicine | Mundada & Shivhare**^2^** |
| 3 | *Amaranthus viridis* | 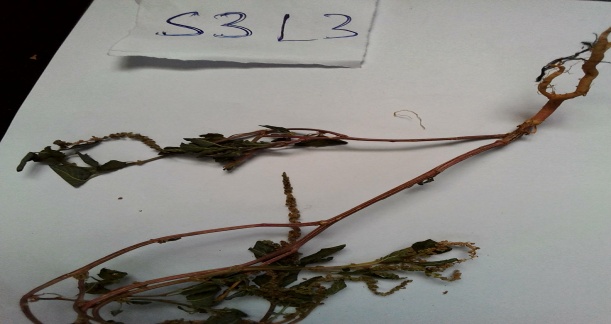 | -Herbal medicine  -Eaten as a boiled green or vegetable  -Used in soup in Nigeria  -Can be eaten by animals | - Reyad-ul-Ferdous *et al***^3^**  -Wikipedia**^4^** |
| 4 | *Acalypha ciliata* | 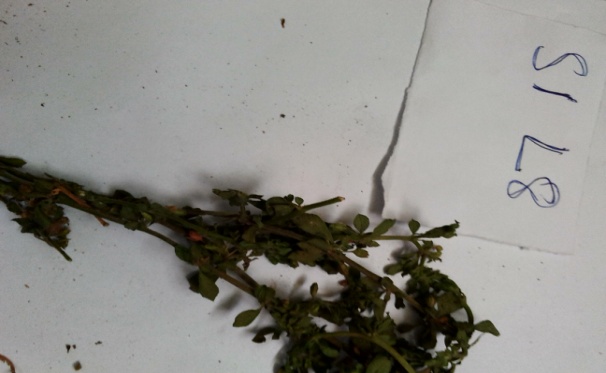 | -Eaten as a vegetable  -Fed to animals  -Herbal medicine  -Bioinsecticide | Schmelzer**^5^** |
| 5 | *Solenostemon monostachyus* | 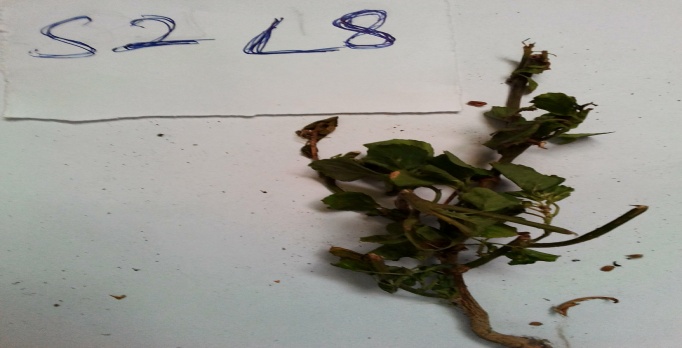 | -Weed  -Medicinal plant  -*Ewé Olójóngbódù* in Yoruba used for high blood pressure  -Leaf extract is a good inhibitor of aluminium corrosion in acidic medium | Abakedi**^6^** |
| 6 | *Scoparia dulcis* | 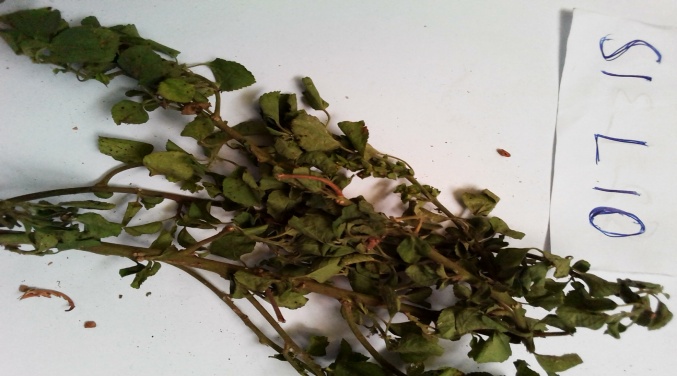 | -Weed  -Medicinal herb  -Antidote for snakebites and cassava intoxication  -Fresh or dried plants used to kill fleas, lice and intestinal worms | Ken Fern**^7^** |
| 7 | *Ferns* | 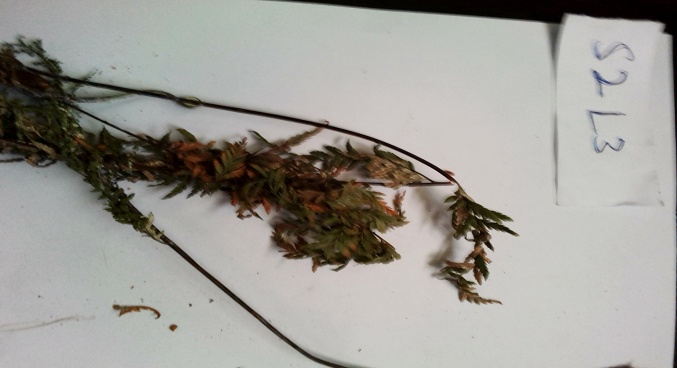 | -Food  -Medicine  -Biofertilizer  -Ornamental plants  -Remediation of contaminated soil  -Able to remove some chemical pollutants from the atmosphere | Wikipedia**^8^** |
| 8 | *Kyllinga sp* | 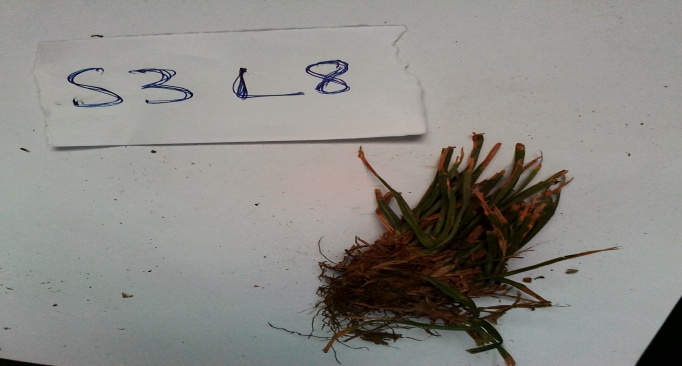 | -Weed  -Foraged plant  -Herbal medicine | -Bryson *et al***^9^**  -Sindhu *et al***^10^** |
| 9 | *Microgramma owariensis* | 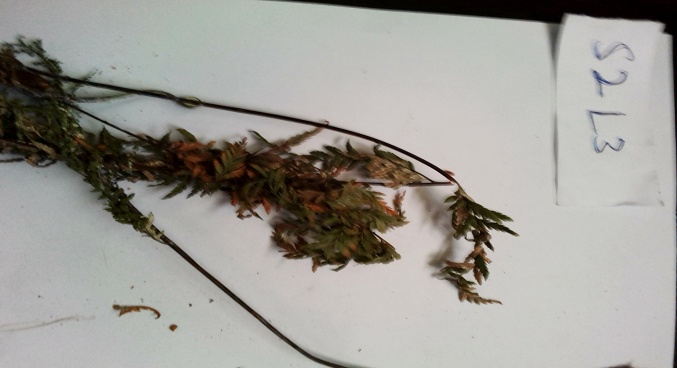 | -Weed  -Medicinal |  |

**References**

**1. Gusau LH, Ogbiko C, Msheila HE, Umar AU, and Umar KJ**. Phytochemical and Antioxidant Study of Different Solvents Extract of Monechma Ciliatum (Acanthaceae): An In Vitro Study. International Journal of Innovative Research and Advanced Studies (IJIRAS). 2018 5(5): 222-225. Available from: <https://www.ijiras.com/may-issue-2018>

**2. Mundada, S. and Shivhare, R**. Pharmacology of *Tridax procumbens* a Weed: Review. International Journal of PharmTech Research CODEN (USA). 2010. 2 (2): 1391-1394

**3. Reyad-ul-Ferdous, Md., Shahjahan, DMS, Tanvir, S, and Mukti, M**. Present Biological Status of Potential Medicinal Plant of *Amaranthus viridis*: A Comprehensive Review. American Journal of Clinical and Experimental Medicine, 2015. 3(5-1): 12-17. <https://doi.org/10.11648/j.ajcem.s.2015030501.13>

**4. Wikipedia.** *Amaranthus viridis*. last edited on 30 September 2020, at 22:57 (UTC). Accessed [2020 May 10] https://en.wikipedia.org/wiki/Amaranthus_viridis

**5. Schmelzer, GH.** *Acalypha ciliata* Forssk. [Internet] Record from PROTA4u Schmelzer, GH & Gurib-Fakim, A (Editors). PROTA (plant Resources of Tropical Africa/ Resources Vegetales de l’Afrique tropicale), Wageningen, Netherlands. Accessed [2020 May 10] Available at: <https://www.prota4u.org/database/protav8.asp?g=pe&p=Acalypha+ciliata+Forssk>.

**6. Abakedi, OU.** Effect of *Solenostemon monostachyus* Leaf Extract on Mild Steel Corrosion in H_2_SO_4_ Solution. International Journal of Innovative Environmental Studies Research. 2017. 5(2):10-16. https://seahipaj.org/index.php/journals/engineering-technology-and-environment/ijiesr/vol-5-issue-2/

**7. Ken Fern.** *Scoparia dulcis*. Tropical Plants Database, tropical.theferns.info. 2020-10-05.
Updated [2019 June 13] Accessed [2020 October 5] Available from: <http://tropical.theferns.info/viewtropical.php?id=Scoparia+dulcis>.

**8. Wikipedia.** Ferns (disambiguation) Accessed [2020 October 5] <https://en.wikipedia.org/wiki/Ferns_(disambiguation)>

**9.**  **Bryson, CT., Carter, R., McCarty, LB. and Yelverton, F.** Kyllinga, a Genus of Neglected Weeds in the Continental United States. Weed Technology, 1997.11(4): 838-842

**10. Sindhu, T., Rajamanikandan, S., & Srinivasan, P.** In vitro Antioxidant and Antibacterial Activities of Methanol Extract of Kyllinga nemoralis. Indian Journal of Pharmaceutical Sciences, 2014.76(2): 170–174. DOI: 10.4103/0250-474X.131540 . https://www.ijpsonline.com/articles/iin-vitroi-antioxidant-and-antibacterial-activities-of-methanol-extract-of-ikyllinga-nemoralisi.pdf
